# Predictors of HIV testing among youth 15–24 years in urban Ethiopia, 2017–2018 Ethiopia population-based HIV impact assessment

**DOI:** 10.1371/journal.pone.0265710

**Published:** 2023-07-19

**Authors:** Aderonke S. Ajiboye, Frehywot Eshetu, Sileshi Lulseged, Yimam Getaneh, Nadew Tademe, Tsigereda Kifle, Rachel Bray, Hailegnaw Eshete, Yohannes Demissie, Clare A. Dykewicz, David Hoos, EPHIA Study Group

**Affiliations:** 1 Division of Global HIV and TB, Center for Global Health, CDC, Addis Ababa, Ethiopia; 2 ICAP at Columbia Universfity, Addis Ababa, Ethiopia; 3 Ethiopia Public Health Institute, Federal Ministry of Health of Ethiopia, Addis Ababa, Ethiopia; 4 ICAP at Columbia University, New York, New York, United States of America; University of Zimbabwe Faculty of Medicine: University of Zimbabwe College of Health Sciences, ZIMBABWE

## Abstract

**Introduction:**

Youth (adolescents and young adults) aged 15–24 years comprise approximately 22% of Ethiopia’s total population and make up 0.73% of HIV cases in urban Ethiopia. However, only 63% of HIV-positive youth are aware of their HIV status. We describe the HIV testing behaviors of youth 15–24 years and determined the characteristics of those who were most likely to be tested for HIV within the past year.

**Methods:**

Using data from the 2017–2018 Ethiopia Population-based HIV Impact Assessment, we provide survey-weighted estimates and prevalence risk ratios for engagement in HIV testing in the 12 months preceding the survey. We model the likelihood of HIV testing one year or more before the survey compared to never testing, using a multinomial logistic regression model.

**Results:**

Among HIV-negative and unaware HIV-positive youth 15–24 years old (N = 7,508), 21.8% [95% Confidence Interval (CI): 20.4–23.3%] reported testing for HIV in the last 12 months. Female youth [Prevalence Ratio (PR) = 1.6, 95% CI: 1.4–1.8], those aged 20–24 years (PR = 2.6, 95% CI:2.3–2.9), and those ever married (PR = 2.8, 95% CI: 2.5–3.1) were more likely to have tested for HIV within the last year. Adjusting for select demographic characteristics, sex with a non-spousal or non-live-in partner [Relative Risk (RR) = 0.3, 95% CI:0.1–0.8] among males did not increase their likelihood to test for HIV in the prior 12 months. Female youth engaged in antenatal care (RR = 3.0, 95% CI: 1.7–5.3) were more likely to test for HIV in the past year.

**Conclusion:**

The Ethiopian HIV case finding strategy may consider approaches for reaching untested youth, with a specific focus on adolescent males,15–19 years of age. This is critical towards achieving the UNAIDS HIV testing goal of 95% of all individuals living with HIV aware of their status by 2030.

## Introduction

Since 1990, under-five child mortality in Eastern and Southern Africa has steadily decreased by 3.7% each year [1]. The reduction in child mortality over the last 30 years in sub-Saharan Africa (SSA) has contributed to what is known today as the African ‘youth bulge’, with 60% of the total sub-Saharan African population under the age of 25 years [2]. The rapidly increasing youth population across sub-Saharan Africa has introduced new challenges in the fight to end HIV/AIDS. Historically, perinatally infected children were assumed to not have survived past childhood. However, recent studies in Zimbabwe and South Africa have shown that an increasing number of undiagnosed adolescents are now presenting at primary care facilities with symptoms of long-term HIV infection [3, 4]. Many new cases of HIV infection are also occurring within the 15–24-year-old age group. As of 2019, those 15–24-years-old made up 36% of all new HIV infections among all adults 15 years and older in sub-Saharan African [5]. In 2017 alone, an estimated 290,000 new HIV infections occurred among those 15–24 years in Eastern and Southern Africa with two-thirds of these infections among young women [2]. If not for the youth bulge, it is estimated that between 2010–2017, there would have been 340,000 fewer new cases of HIV among sub-Saharan African youth, aged 15–24 years [2].

The Joint United Nations Programme on HIV/AIDS (UNAIDS) recommends a 90-90-90 treatment target for ending the HIV epidemic by the year 2020: 90% of all people living with HIV to be aware of their HIV-positive status, 90% of HIV-positive individuals receiving HIV treatment, 90% of HIV-treated individuals achieving HIV viral-load suppression [6]; these targets were updated to 95-95-95 to be achieved by the year 2030 [6]. A pooled analysis of 2016 population-based HIV surveillance data from Malawi, Zambia, Zimbabwe found that only 46% of all HIV-positive youth 15–24 years were aware of their HIV-positive status [6, 7]. Saharan African youth are achieving less than half of the recommended 1st 90 target and falling behind older adults, who are 78% aware of their current HIV-positive status [7]. Among youth aware of their status, 82% received HIV treatment, and 79% of these achieved HIV viral load suppression. The 2016 analysis shows that for older adults, 90% of those aware received HIV treatment and 90% of those on treatment achieved HIV viral load suppression [7]. As the youth population continues to grow across sub-Saharan Africa, the greatest challenge to reaching HIV epidemic control will be the identification of new and untreated cases of HIV.

In Ethiopia, Africa’s second most populous country, only 63% of HIV-positive youth 15–24 years are aware of their HIV-positive status [8]. Among those aware, 100% were on antiretroviral HIV treatment, and 78.6% of those on treatment had achieved HIV viral load suppression [8]. Ethiopian youth make up 22% of the nation’s total population [9], and as of 2018, the prevalence of HIV amongst this age group was 0.7% [8]. Nationally, the HIV epidemic in Ethiopia is concentrated in the urban and more densely populated parts of the country, with an urban HIV prevalence of 4.2%, which is almost triple the national prevalence of 1.5% [9]. The prevalence of HIV in Ethiopia is lower than that of neighboring countries such as Kenya (4.9%) and Uganda (5.3%) [10]. Despite having a low prevalence of HIV, Ethiopian youth are well below the first UNAIDS recommended target of 90% awareness of HIV positive status [11]. Additionally, comprehensive knowledge of HIV transmission and prevention methods among youth is low. According to the 2016 Ethiopia Demographic Health Survey (EDHS), only 24% of women and 39% of men 15–24 years of age possessed a comprehensive knowledge of HIV prevention methods—which includes the understanding that a healthy-looking person can have HIV, and understanding that sexual intercourse with one, uninfected partner and consistent condom-use helps to reduce the risk of acquiring HIV [12].

The Ethiopia national HIV program has implemented several strategies to increase efforts to identify untreated cases of HIV. These strategies mainly employ provider-initiated case finding and social networking testing [13]. The services offered within these strategies, index case testing and partner notification, have historically targeted the married partners and biological children of HIV-positive individuals [13]. The national program also recommends the availability of youth-friendly HIV testing and counseling (HTC) services; however, the nature of these youth-friendly services is not clearly defined. With only 63% of HIV-positive youth in Ethiopia aware of their status, the current national HIV testing strategies may be missing undiagnosed, HIV-positive youth. This paper seeks to identify the demographic and behavioral characteristics of the Ethiopian youth 15–24-year-old age group, as well as the aspects of the current healthcare system, that facilitate the engagement of Ethiopian youth into HIV testing. Within a country with an increased incidence of HIV, adequate access to HTC is needed to achieve national HIV epidemic control; this evaluation is needed to understand where gaps in HIV testing coverage exists. The goal of these findings is to assist the Ethiopia national HIV program to identify strategies to scale-up youth-friendly HIV testing services, to achieve 95% awareness of HIV-positive status within the youth age group.

## Methods

### Study design and population

This paper is a secondary analysis of the 2018 Ethiopia Population-based HIV Impact Assessment (EPHIA). The EPHIA was a cross-sectional, household-based sero-survey conducted in urban Ethiopia from October 2017 to March 2018. The survey aimed to estimate the prevalence of HIV and the coverage of HIV prevention and treatment services among adults and adolescents between 15–64 years. Our analysis includes eligible household members between the ages of 15–24 years old, who slept in the household the night before the survey, spoke one of six survey languages and were willing and able to provide written informed consent or assent. Non-de facto household members, those who did not sleep in the household the night before the survey, were not eligible for participation. We assessed the HIV testing behaviors of persons aged 15–24 years and focused on those who were HIV-negative or unaware of their HIV-positive status. We aim to identify the factors that might facilitate youth’s access to HIV testing, particularly among those at risk of acquiring HIV and those at risk of unknowingly transmitting the virus.

### Sampling design

The EPHIA used a two-stage cluster sampling design [14] to select a representative sample of enumeration areas (EAs) and households across urban areas of Ethiopia. Urban was defined as any region, district, or capital where residents do not engage in agricultural practices as the primary source of income. The 2007 National Ethiopian Census served as the sampling frame for the selection of enumeration areas. The first stage of sampling included 393 EAs selected randomly from all eleven regions of the country based on a probability proportional to regional population size. The second stage of sampling involved an average number of 30 households randomly selected per cluster, using an equal probability method, for a total of 11,810 households.

### Participant eligibility and data collection

A household questionnaire was administered to selected households. The questionnaire assessed for the biobehavioral factors related to HIV infection, including sociodemographic factors, reproductive history, sexual activity, HIV testing and the utilization of HIV care and treatment services, and attitudes towards HIV disclosure. Data from household and individual interviews were collected using mobile tablets and were stored on a central server [15].

All eligible participants received home-based HIV testing and counseling (HBTC) following Ethiopia’s rapid HIV diagnostic algorithm. Although participants self-reported their HIV status in the individual questionnaire, HIV status for the survey was defined by the result of the HIV rapid test. HIV rapid test results were returned to consenting participants at the household. Laboratory confirmation of seropositive samples was conducted using the Geenius HIV-1/2 supplemental assay. Self-reported awareness of HIV-positive status was confirmed by HIV-1 RNA and antiretroviral (ARV) drug testing [16].

### Variables

We assessed the demographic, behavioral, and structural factors associated with HIV testing in the year preceding the survey. We generated a three-level outcome variable comparing: 1) those who self-report testing in the 12 months preceding the survey, 2) those self-reporting testing more than 12 months since the survey, and 3) those who never tested. Those who reported ever testing for HIV and reported testing in the 12 months prior to the survey, regardless of whether they received their test results, were categorized as having tested for HIV in the last 12 months. We categorized those reporting a last HIV test date as more than 12 months preceding the survey separately. Those who did not report ever testing for HIV were categorized as having never tested.

Age, marital status, education, employment, and urban area size are covariates in this analysis. Youth were disaggregated into two five-year age groups of 15–19 years and 20–24 years. For this analysis, the term ‘adolescent’ refers to persons aged 15–19 years, the term ‘young adult’ are persons aged 20–24 years, and the term ‘youth’ describes all those aged 15–24 years. Small and large urban areas were determined by their population size. Small urban areas were those with less than 50,000 people and large urban areas were areas with 50,000 people or more. Individuals missing data due to a response of “don’t know” or refusal to answer the questions on marital status, education, and employment were categorized with those who answered negatively to the question (never married, no education, and not employed, respectively). Those missing a response were included in the sample to maintain the overall sample size and the power within the regression models. Of this sample, 0.7% of respondents are missing or did not disclose their marital status, 0.1% of respondents did not disclose their employment status, and 0.2% of respondents are missing or did not disclose their highest level of education.

The sexual risk behaviors included in this analysis were age at first sex, the presence of a non-spousal or non-live-in sex partner in the past 12 months, condom use at last sex in the past 12 months, and engagement in transactional sex. To evaluate the accessibility of HIV testing within the Ethiopian healthcare system, we included the EPHIA variables for antenatal care (ANC) and attendance at tuberculosis clinics in our analysis.

### Data analysis

Survey weights were applied to the responses to the household interview, the individual participant interview, and the HIV biomarker survey data. Sampling weights allow for two-cluster survey data to be generalizable beyond the sample by accounting for sample selection probability and for the nonresponse of the households and individuals included in the original sample. We analyzed responses from survey participants ages 15–24 years who had complete questionnaire and HIV testing data, and thus used the biomarker weights. Additionally, data analysis was only conducted among HIV-negative and unaware HIV-positive individuals, with awareness of HIV status confirmed through self-reporting or confirmatory ARV drug testing. Individuals that were found to have ARVs in their blood were considered aware of their HIV status and excluded from the analysis.

We generated bivariate weighted percentages and prevalence ratios (PRs) of testing for HIV in the last 12 months by select demographic, behavioral, and healthcare covariates. We conducted significance testing (p<0.05) to determine if prevalence ratios for testing for HIV in the last 12 months significantly differed from 1, and generated p-values (Table 2). We generated two multinomial logistic regression models [17], separating male and female respondents to eliminate the bias of confounding by gender. In the multinomial model, we compared the likelihood for testing in the past 12 months and testing more than 12 months before the survey to the likelihood of never testing. The models were adjusted for age, marital status, and employment in the past 12 months. To assess sexual behavioral risk for HIV infection, the variable for ‘non-spousal sex partner in the past 12 months’ was included in the final model. Similarly, medical male circumcision and antenatal care attendance served as indicators for healthcare access within their respective male and female multivariate models. We conducted significance testing to determine if the adjusted relative risk ratios significantly differed from 1, using a p-value of less than 0.05. Stata statistical software version SE 15.0 was used to perform all statistical analysis. All weighted estimates and 95% confidence intervals (CI) were calculated using jackknife replicate weights that account for sampling, nonresponse, and under coverage in the variance estimation.

### Ethics for human subjects research

The study was approved by the Institutional Review Boards at the Ethiopia Public Health Institute, Columbia University Medical Center, Westat, and the Institutional Review Boards at The Centers for Disease Control and Prevention in Atlanta and Ethiopia. The survey was conducted by the Ethiopia Public Health Institute (EPHI), the Central Statistics Agency of Ethiopia, ICAP at Columbia University, Westat, and with technical assistance provided by the CDC. Participant consent was obtained through written informed consent or assent. Adult participants 18–64 years and emancipated minors 13–17 years provided informed consent prior to completing the individual interview and prior to survey blood collection. Adolescents 12–17 years were required to obtain parental permission and provide written assent to participate in the interview and blood collection.

## Results

**Fig 1:**
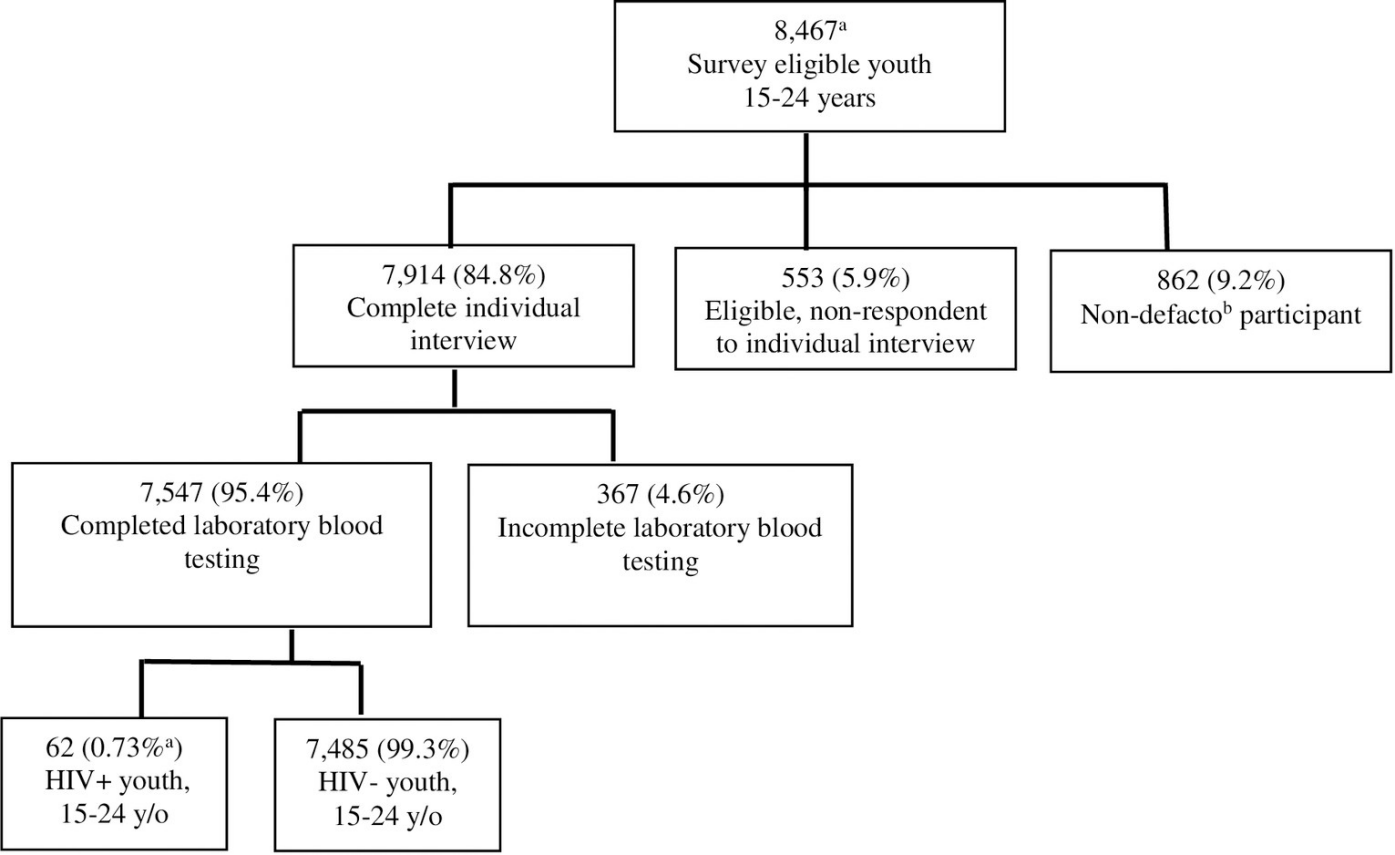
Survey interview completion and the results of laboratory HIV testing among youth, 15–24 years old, Ethiopia -based HIV impact assessment 2017–2018. **(**a) Frequencies and percent estimates are unweighted. (b) The EPHIA defines a non-defacto participant as a usual household member who did not sleep in the household the night before the survey.

**Fig 2:**
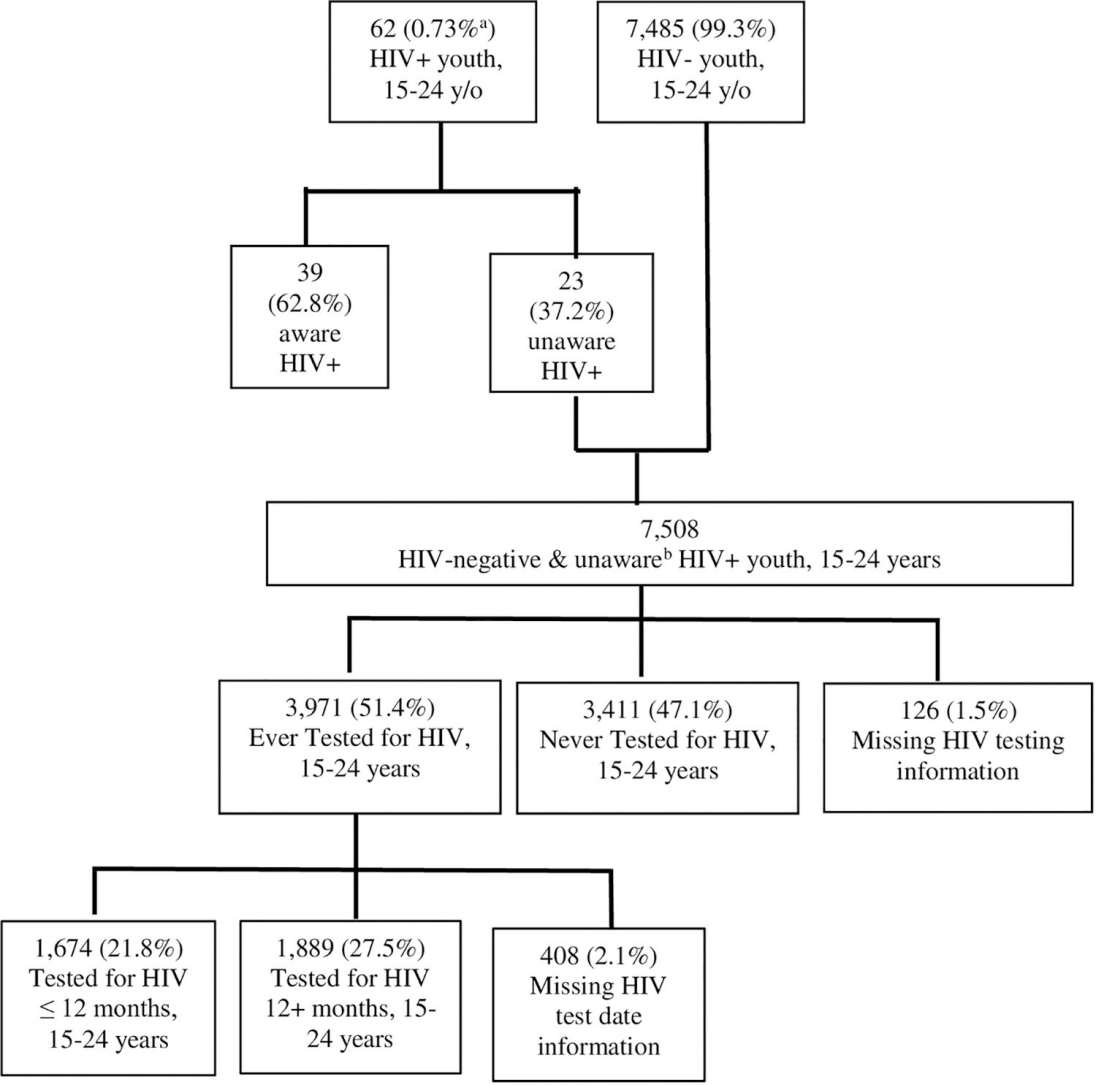
Engagement in HIV testing among HIV-negative and unaware HIV-positive youth, 15–24 years old, Ethiopia population-based HIV impact assessment 2017–2018. Flowchart of youth engagement in HIV testing prior to EPHIA survey participation among HIV-negative and unaware HIV-positive youth, aged 15–24 years. (a) percent estimates are weighted using jackknife survey replicate weights. (b) awareness of HIV status was confirmed via participant self-report of HIV status and HIV antiretroviral metabolite testing.

A total of 7,547 youth aged 15–24 years were interviewed from selected households and completed HIV testing (Fig 1). Of these, 0.7% (n = 62) tested HIV positive; 37.2% (n = 23) of those positive were confirmed to be unaware of their HIV-positive status. Our analysis examines those 7,382 youth who tested negative for HIV or were found to be unaware of their HIV-positive status and had complete HIV testing information (Fig 2). Overall, 49.6% (95% CI: 49.4–49.7, n = 3,805) of all respondents were 15–19 years old and 50.5% (95% CI: 50.3–50.6, n = 3,577) were 20–24 years old, and 49.8% (95% CI: 49.6–50.0) of respondents were male (Table 1).

**Table 1:** Demographic characteristics, sexual risk behaviors, and health seeking behaviors of HIV-negative and unaware HIV-positive youth 15–24 years in urban Ethiopia, EPHIA 2017–2018.

	Male		Female		Total Percentage	
	n	% (95 CI)	n	% (95 CI)	N	% (95 CI)
**Total (15–24 years)**	2, 696	49.8 (49.6–50.0)	4,686	50.2 (50.1–50.4)	7,382	100
***Demographic Characteristics***						
Age group						
**15–19**	1,425	49.4 (49.1–49.7)	2,380	49.7 (49.5–49.9)	3,805	49.6 (49.4–49.7)
**20–24**	1,271	50.6 (50.3–50.9)	2,306	50.3 (50.1–50.5	3,577	50.5 (50.3–50.6)
Urban Area size						
**Small (≤50,000)**	1,456	54.9 (48.6–61.0)	2,232	48.1 (42.6–53.6)	3,688	51.5 (45.9–57.0)
**Large (>50,000)**	1,240	45.1 (39.0–51.4)	2,454	51.9 (46.4–57.4)	3,694	48.5 (43.0–54.2)
Marital Status						
**Married**	277	11.1 (9.3–13.2)	1,591	33.3 (31.0–35.6)	1,868	22.3 (20.5–24.1)
**Never Married/missing**	2,398	88.9 (86.8–90.7)	3,065	66.7 (64.4–69.0)	5,463	77.8 (75.9–79.4)
Education Status						
**No Education**	69	2.7 (2.0–3.6)	236	4.6 (3.8–5.6)	305	3.6 (3.1–4.3)
**Primary**	834	30.3 (27.5–33.3)	1,637	34.3 (32.4–36.3)	2,471	32.3 (30.4–34.3)
**Secondary/Post-Secondary**	1,793	67.0 (63.8–70.0)	2,813	61.1 (58.8–63.3)	4,606	64.0 (61.8–66.2)
Employment (in past 12 months)						
**Yes**	890	34.7 (31.6–37.9)	1,190	26.5 (24.9–28.2)	2,080	30.6 (28.7–32.5)
**No/ Missing/ DK**	1,806	65.3 (62.1–68.4)	3,496	73.5 (71.8–75.2)	5,302	69.4 (67.5–71.3)
***Sexual Risk Factors***						
Sex Ever						
**Ever had sex**	875	34.3 (32.0–36.7)	2,101	44.8 (42.6–47.0)	2,976	39.6 (37.8–41.4)
**Never had sex/Missing**	1,786	65.7 (63.4–68.0)	2,510	55.2 (53.1–57.4)	4,296	60.4 (58.6–62.2)
Among those reporting sex, sex before age 15						
**Yes**	134	13.4 (11.1–16.1)	395	18.7 (16.5–21.0)	529	16.4 (14.7–18.2)
**No**	741	86.6 (83.9–88.9)	1,706	81.3 (79.0–83.5)	2,447	83.6 (81.8–85.3)
Non-spousal/ non-live in sexual partner in the past 12 months						
**Yes**	331	58.1 (52.4–63.5)	351	23.0 (20.2–25.9)	682	36.8 (33.5–40.3)
**No**	227	41.9 (36.5–47.6)	1,199	77.1 (74.1–79.8)	1,426	63.2 (59.8–66.5)
Condom use at last sex (<12 months)						
**Yes**	175	30.7 (25.3–36.6)	119	7.8 (6.4–9.4)	294	16.6 (14.3–19.1)
**No condom at last intercourse**	351	69.3 (63.4–74.7)	1,400	92.2 (90.6–93.6)	1,751	83.4 (80.9–85.7)
STD Diagnosis						
**Yes**	8	0.7 (0.3–1.7)	30	1.5 (1.0–2.2)	38	1.2 (0.8–1.7)
**No**	902	99.3 (98.3–99.7)	2,145	98.5 (97.8–99.0)	3,047	98.8 (98.3–99.2)
***Health Seeking Behaviors***						
Medical Male Circumcision						
**Yes**	741	30.1 (26.5–33.9)				
**No/DK/Refused**	1,955	69.9 (66.1–73.5)				
ANC attendance among women giving birth in last three years						
**Yes**			576	94.5 (89.8–97.1)		
**No**			30	5.5 (2.9–10.2)		
Ever visited a TB clinic						
**Yes**	69	2.9 (2.1–4.0)	102	2.1 (1.7–2.6)	171	2.5 (2.0–3.1)
**No**	2,627	97.1 (96.0–97.9)	4,584	97.9 (97.4–98.3)	7,211	97.5 (96.9–98.0)

^a^Percent estimates are weighted using jackknife survey replicate weights

^b^Awareness of HIV status was confirmed via participant self-report of HIV status and ARV testing

### Sample characteristics

Youth participants resided, almost evenly, between small and large urban areas (Table 1). Twenty-two percent (22.3%, 95% CI: 20.5–24.1) of all youth have ever married or lived with a partner as if married (Table 1). However, the proportion of female youth who have married was three times that of male youth. Among urban youth 15–24 years, 64% (95% CI: 61.8–66.2) report achieving secondary or post-secondary education as their highest level of education. More than two-thirds (69.4%, 95% CI: 67.5–71.3) of all youth were not employed or did not receive compensation for work in the 12 months preceding the survey (Table 1).

Nearly forty percent (39.6%, 95% CI: 37.8–41.4) of all youth 15–24 years reported ever having sex. Of these, 18.7% (95% CI: 16.5–21.0) of females and 13.4% (95% CI: 11.1–16.1) of males report sexual debut before the age of 15. Thirty-seven percent (36.8%, 95% CI: 33.5–40.3; n = 682) of youth reported having at least one non-spousal or non-live-in sexual partner in the last year; but the rate was higher in males (58.1%, 95% CI: 52.4–63.5) versus females (23.0%, 95% CI: 20.2–25.9). For youth who reported having sex in the 12 months preceding the survey, 83.4% (95% CI: 80.9–85.7; n = 1,751) reported that they did not use a condom at last sex. Proportionally more female than male youth reported not using a condom at last sex (92.2% (95% CI: 90.6–93.6) vs. 69.3% (95% CI: 63.4–74.7), respectively).

Thirty percent (30.1%, 95% CI: 26.5–33.9) of male youth had a medical circumcision (circumcision performed by a physician or clinical officer) (Table 1). Among female youth who gave birth in the three years preceding the survey, 94.5% (95% CI: 89.8–97.1) report to have received antenatal care during their most recent pregnancy (Table 1).

### Prevalence of HIV testing in the past 12 months

Among HIV-negative and HIV-positive Ethiopian youth unaware of their HIV status, 51.4% (n = 3,971) report ever being tested for HIV (Fig 2). Twenty-two percent (21.8%) of these youth reported to having an HIV test in the 12 months preceding the survey and 27.5% tested for HIV more than a year before the survey (Fig 2). Proportionally more youth aged 20–24 than youth 15–19 years reported having tested in the last 12 months (31.4% (95% CI: 29.2–33.7) vs. 12.3% (95% CI: 10.9–13.8), respectively (Table 2). Twenty-seven percent (26.8%, 95% CI: 25.2–28.6) of female youth reported testing for HIV in the last 12 months, while only 16.8% (95% CI: 14.8–19.0) of male youth have tested within this same timeframe. Forty-seven (47.6%, 95% CI: 42.5–52.8) percent of female youth who attended an antenatal clinic in the last three years also received an HIV test in that year (Table 2). Forty percent, (40.5%, 95% CI: 32.8–48.8) of those who have ever been to a TB clinic also received HIV testing in the last year (Table 2).

**Table 2:** Percentage^†^ and prevalence ratio of testing for HIV ≤12 months among HIV-negative and unaware HIV-positive youth (15–24 years), by demographic characteristics, EPHIA 2017–2018.

Characteristics	Tested for HIV in past 12 months		N	Prevalence Ratio	P-value
	Yes (%)	95% CI		PR (95% CI)	
**Total (15–24 years)**	21.8	20.4–23.3	6, 974	--	---
***Demographics***					
**Age group**					
15–19	12.3	10.9–13.8	3,647	*ref*	0.00
20–24	31.4	29.2–33.7	3,327	2.6 (2.3–2.9)	
**Gender**					
Male	16.8	14.8–19.0	1,892	*ref*	0.00
Female	26.8	25.2–28.6	3,193	1.6 (1.4–1.8)	
**Urban Area size**					
Small (≤50,000)	20.5	18.6–22.6	3,469	*ref*	0.09
Large (>50,000)	23.2	20.9–25.5	3,505	1.1 (1.0–1.3)	
**Marital Status**					
Never Married/DK/Refused	15.7	14.3–17.3	5,203	*ref*	0.00
Married/Living Together	43.9	40.8–47.1	1,721	2.8 (2.5–3.1)	
**Education Status**					
No Education	24.9	(18.5–32.7)	285	*ref*	
Primary	19.3	(17.7–21.0)	2,336	0.77 (0.58–1.0)	0.07
Secondary/Post- Secondary	22.9	(21.0–24.8)	4,353	0.92 (0.69–1.2)	0.54
**Employment (in past 12 months)**					
No	18.5	(17.2–20.0)	5,037	*ref*	0.00
Yes	29.4	(26.8–32.0)	1,937	1.6 (1.4–1.8)	
***Sexual Risks***					
**Sex Ever**					
Never had sex/missing	11.9	(10.5–13.4)	4,114	*ref*	0.00
Ever had sex	37.7	(35.2–40.2)	2,752	3.2 (2.8–3.6)	
**Condom last sex (past 12 months)**					
No condom/ No Sex	42.5	(39.4–45.6)	1,601	*ref*	0.04
Yes	33.9	(27.0–41.5)	263	0.80 (0.60–1.0)	
**Non-spousal/ non-live in sexual partner in past 12 months**					
No	44.9	(41.3–48.6)	1302	*ref*	0.00
Yes	33.0	(28.6–37.8)	616	0.74 (0.63–0.9)	
**STD Diagnosis in the past 12 months**					
No	36.8	(34.3–39.3)	2,824	*ref*	0.04
Yes	54.3	(35.4–72.1)	35	1.5 (1.0–2.1)	
***Health Seeking Behaviors***					
**Medical Male Circumcision**					
Non-medical	22.9	(21.5–24.4)	6,263	*ref*	0.00
Medical	15.6	(11.9–20.1)	711	0.7 (0.50–0.90)	
**ANC Attendance among women giving birth in last three years**					
No	24.7	(8.9–52.3)	30	*ref*	0.19
Yes	47.6	(42.5–52.8)	525	1.9 (0.70–5.3)	
**Ever visited a TB clinic**					
No/DK/Missing	21.4	(19.9–22.9)	6,827	*ref*	0.00
Yes	40.5	(32.8–48.8)	147	1.9 (1.5–2.4)	

^a^Percent estimates were weighted using jackknife survey replicate weights

^b^Awareness of HIV status was confirmed via participant self-report of HIV status and ARV metabolite testing

^c^Prevalence ratio significance is at p<0.05

### Predictors of HIV testing in youth

For all youth, age, gender, marital status, and recent employment were significant predictors of HIV testing in the 12 months preceding the survey. Young adults (20–24 years) were twice as likely to have tested for HIV in the last 12 months than those 15–19 years of age (PR = 2.6, 95% CI: 2.3–2.9) (Table 2). Regardless of age, female youth were almost twice as likely to have tested in the last year in comparison to male youth (PR = 1.6, 95% CI: 1.4–1.8). Those ever married or have lived with a partner were three times as likely to have tested in the last 12 months (PR = 2.8, 95% CI: 2.5–3.1). Employed youth were nearly twice as likely to have HIV tested in the past year than unemployed youth (PR = 1.6, 95% CI: 1.4–1.8).

Youth who have been sexually active (PR = 3.2, 95% CI:2.8–3.6), and those who received an STD diagnosis in the last year (PR = 1.5, 95% CI: 1.0–2.1) were significantly more likely to have tested for HIV in the last year than to have never tested (Table 2). Those reporting at least one non-spousal, non-live in sexual partner in the past 12 months were significantly less likely to have tested for HIV in the past year, in comparison to those with all marital or live-in partners (PR = 0.74, 95% CI: 0.6–0.9). Youth who report using a condom at last sex were significantly less likely to have tested for HIV in the last year (PR = 0.80, 95% CI: 0.6–1.0). Past visits to a TB clinic significantly increased the likelihood of testing HIV in the past 12 months among all youth (Table 2).

Table 3 presents the multinomial logistic regression models for HIV testing by gender. Among males, controlling for variables of sexual behavioral risk and healthcare access, being older (20–24 years) was the only significant predictor for recent HIV testing likelihood. Young adult males (20–24 years), in comparison to adolescent males (15–19 years), had a three-fold likelihood of testing for HIV in the past year (RR = 2.8, 95% CI:1.4–5.7) than never testing. Marital status, education, and history of non-spousal sex partner in the past 12 months were not associated with HIV testing at any time, either in the past 12 months or more than 12 months ago for males (Table 3). In contrast, for female youth, HIV testing in the past 12 months was associated with being older (20–24 years old), being married, having at least a secondary education, and ANC attendance at the last pregnancy (Table 3). This suggests that female youth may receive more effective HIV messages than male youth, and/or that existing HIV services may be more targeted to female youth.

**Table 3:** Among youth ages 15–24 (HIV-negative and unaware HIV-positive), multinomial regression of HIV testing by select demographic characteristics, EPHIA 2017–2018.

Characteristics	Male		Female	
	RR^a^ (95% CI)	P-value	RR (95% CI)	P-value
**Never Tested**	*reference*		*reference*	
**Tested for HIV ≤ 12 months**				
**Age group**				
15–19 (ref)	2.8 (1.4–5.7)	0.0	1.8 (1.2–2.9)	0.0
20–24				
**Urban Area Size**				
Small (≤50,000) (ref)	1.5 (0.8–2.5)	0.2	1.2 (0.7–1.9)	0.5
Large (>50,000)				
**Marital Status**				
Never Married (ref)	1.2 (0.5–2.8)	0.7	2.5 (1.2–5.3)	0.0
Married				
**Employment (in past 12 months)**				
No (ref)	1.2 (0.7–2.2)	0.4	1.2 (0.8–1.8)	0.4
Yes				
**Education Status**				
No Education	reference		reference	
Primary	1.0 (0.3–3.7)	1.0	1.7 (0.8–3.5)	0.2
Secondary/Post- Secondary	2.5 (0.7–9.0)	0.1	3.4 (1.6–7.1)	0.0
**Non-spousal sex partner in the past 12 months**				
No (ref)	0.3 (0.1–0.8)	0.0	1.1 (0.6–2.0)	0.7
Yes				
**Medical Male Circumcision**				
No (ref)	0.6 (0.3–1.2)	0.1		
Yes				
**ANC attendance at last pregnancy**				
No			3.0 (1.7–5.3)	0.0
Yes				
**Tested for HIV >12 months**				
**Age group**				
15–19 (ref)	3.0 (2.0–5.5)	0.0	2.8 (1.7–4.7)	0.0
20–24				
**Urban Area Size**				
Small (≤50,000) (ref)	1.0 (0.6–1.8)	1.0	1.0 (0.7–1.7)	0.8
Large (>50,000)				
**Marital Status**				
Never Married (ref)	2.2 (0.8–5.9)	0.1	2.2 (0.9–5.5)	0.1
Married				
**Employment (in past 12 months)**				
No (ref)	1.0 (0.6–1.7)	0.8	1.0 (0.7–1.5)	1.0
Yes				
**Education Status**				
No Education	reference		reference	
Primary	0.7 (0.3–2.1)	0.3	2.7 (1.2–6.0)	0.0
Secondary/Post- Secondary	1.6 (0.6–4.4)	0.9	5.2 (2.3–11.7)	0.0
**Non-spousal sex partner in the past 12 months**				
No (ref)	0.8 (0.3–2.0)	0.3	0.8 (0.5–1.4)	0.4
Yes				
**Medical Male Circumcision**				
No (ref)	0.9 (0.5–1.6)	0.6		
Yes				
**ANC attendance at last pregnancy**				
No			3.2 (1.8–5.8)	0.0
Yes				

^a^ Relative Risk ratio significance is at p<0.05

## Discussion

Our findings indicate that less than a quarter (22%) of youth 15–24 years in urban Ethiopia have tested for HIV in the past 12 months (Table 2). Older age, female gender, and being in a marital union were each significantly associated with recent HIV testing. When modelled together, for both males and females, older age (20–24 years) was associated with a higher likelihood of testing for HIV in the last year (Table 3). Amongst males only, the risk behavior of sex with a non-marital sex partner in the last year significantly decreased, by 70%, the likelihood of recent HIV testing among male youth. This finding is important, as 58% of male youth report engaging in sex with a non-regular partner in the past 12 months (Table 3). Among female youth, ever being married, having a secondary education or higher and having recently accessed antenatal care all significantly increased the odds of recent HIV testing (Table 3). Overall, our study reveals that the gaps in HIV testing coverage within the 15–24-year-old age group in urban Ethiopia lies among male youth, especially those unmarried and between 15–19 years of age.

### Age and gender differences in HIV testing

The age and sex differences in recent HIV testing among youth in urban Ethiopia are consistent with the results of previous population-based surveys conducted in other Sub-Saharan African countries. A pooled analysis of data from the 2007–2018 Zambia Demographic Health Surveys (DHS) examined age and sex differences in the uptake of HIV testing services among men and women 15–49 years. Women 25–49 years had the greatest increase in HIV testing overtime, HIV testing uptake for older men (25–49 years) and female youth (15–24 years) increased at the same rate, while the uptake of HIV testing increased the least for male youth 15–19 years [18]. In eastern and southern Africa, population-based surveys conducted from 2015–2021 revealed that a quarter of adolescent girls aged 15–19 years tested for HIV in the last year, while only 17% of adolescent boys 15–19 years tested within the same timeframe [19]. These figures were even lower in central and western African countries, with only 13% adolescent girls and 3% of adolescent boys testing within the last year [19]. Our study findings contribute evidence that in sub-Saharan African adolescent boys consistently test for HIV less often than their older and female counterparts. In Ethiopia, female youth can engage in HIV testing as they enter childbearing age, as federal policies promote the integration of HIV testing and counseling into antenatal and prevention of mother-to-child transmission of HIV (PMTCT) services [13]. Male youth do not have a similar entry point. As adolescent boys reach young adulthood, undiagnosed HIV infection may lead to advanced HIV disease or to the unknown spread of HIV to their partners

### Partner testing and non-marital sex partnership

The 2007 Ethiopian Federal Ministry of Health HIV Counseling and Testing (HCT) Guidelines emphasize the promotion of HIV counseling and testing to couples and women [13]. Couples are encouraged to test at ‘pre-engagement, pre-marital, or pre-conception’ stages, with partner notification services offered [13]. While partner testing should be encouraged, our findings show that being in a marital union does not significantly increase HIV testing among males in the 15–24 age group (Table 3). Previous studies have shown that partner-testing services do not necessarily capture men [20, 21]. This was seen in Kenya, where 88% of pregnant women receiving ANC care tested for HIV in the previous year, but only 5% of their male partners had tested within that same timeframe [22]. In Ethiopia, male youth are likely to debut sexually 2.5 years before entering marriage [12]; with males in urban areas marrying even later [12]. Given the proportion of male youth in urban Ethiopia delaying marriage and reporting sex with a non-marital, non-live in partner (Table 1), the Ethiopia national HIV program may consider implementing contract tracing and offering HIV testing for all sexual partners of HIV cases regardless of marital status.

### Effective strategies to increase HIV testing among youth

#### Improve comprehensive HIV Knowledge

Previous studies examining the uptake of HIV testing among male youth in several sub-Saharan African countries have found that having a comprehensive knowledge of HIV significantly increases the uptake of HIV testing among males 15–24 years [18, 23]. Comprehensive knowledge of HIV allows one to assess their individual risk and decide their need for HIV testing. In several sub-Saharan African countries, the challenge has been in disseminating these comprehensive messages to youth. Currently, the Ethiopia national HIV program supports the dissemination of youth-specific HIV education materials. Formally disseminating these materials through a radio, newspaper, or television media campaign may also be effective, as these mediums have proven to be effective at reaching urban male youth [23].

#### Home-based HIV self-test kits

A community-based approach to HIV testing through the distribution of at-home self-test kits may be an effective strategy to increase access to HIV testing for adolescent males in urban Ethiopia. In communities Zambia and South Africa that achieved 90% awareness of HIV status among female youth, the door-to-door distribution of home-based self-test kits in select communities was effective at increasing the uptake of HIV testing and awareness of HIV status among males 16–29 years [24]. In Malawi, where HIV prevalence in youth is relatively low in comparison to adults, an index-testing strategy to test the children of HIV-positive adults through home-based testing yielded a 94% uptake of HIV testing among children <15 years and youth 15–24 years [25]. Evidence-based differentiated service delivery models have utilized community healthcare workers and teen-based Saturday-clinics to deliver HIV treatment to adolescents living with HIV [26]. Similar strategies for the distribution of at-home self-test kits could be explored in urban settings to reach adolescents and males who are not likely to seek facility-based testing.

The results of our analysis uniquely differ from much of previous research on adolescent and young adult HIV testing in Africa. In an analysis of DHS data from four sub-Saharan African countries, (Nigeria, Uganda, Mozambique, and Congo) males and those ages 15–19 years were less likely to have tested for HIV in comparison to their older, female counterparts [27]. Our inclusion of male youth, 15–24, highlight an additional demographic for which HIV testing services should be targeted. Additionally, our analysis examines the engagement of HIV testing within the last 12 months. Examining HIV testing with this temporal restriction allows for a more accurate evaluation of the reach of current HTC strategies.

### Limitations

The implications of our analysis are limited by the nature in which the data were collected. As a household survey, HIV testing history was captured through self-report rather than from an electronic medical record. Our data is subject to recall bias as respondents may have forgotten if they have ever tested or misreported the date of their last HIV test. The data is also subject to social desirability bias. The age of sexual consent in Ethiopia is eighteen [15]. Youth younger than 18 years of age may not disclose their prior sexual activity or engagement in HIV testing services to avoid any cultural stigma associated with engaging in sex before the age of consent. Estimates for testing for HIV and sexual risk behavior are likely underreported as a result. Lastly, the inclusion of unemancipated minors (15–17 years) in our sample required that they be related to an eligible and consenting adult. Our findings may better characterize the HIV testing behaviors of emancipated minors, 15–17 years old. Emancipation status was not included as a possible predictor of testing due to our sample including youth who are and are not of legal age.

## Conclusion

In urban Ethiopia, adolescents, aged 15–19 years, and males are not being adequately reached within the national HIV counseling and testing strategy in urban Ethiopia. Adolescent girls have a high probability of obtaining testing services if pregnant, through the promotion of HIV testing services in antenatal care. Our findings show that adolescent males are likely to be left out of the existing Ethiopia’s targeted HIV testing strategy.

Among 15–24 years old males who report recent sex, most report sex outside of a committed partnership and few report consistent use of HIV prevention methods. Efforts to increase comprehensive knowledge of HIV and expand access to HIV testing, through at-home, self-test kits have all proven to be effective at reaching adolescent males in national HIV testing efforts. Expanding current HIV testing efforts to include male youth friendly services is important for the future of HIV epidemic control in urban Ethiopia.
